# Exploring Bacterial and Fungal Biodiversity in Eight Mediterranean Olive Orchards (*Olea europaea* L.) in Tunisia

**DOI:** 10.3390/microorganisms11041086

**Published:** 2023-04-21

**Authors:** Houda Gharsallah, Ines Ksentini, Olfa Frikha-Gargouri, Karama Hadj Taieb, Haifa Ben Gharsa, Christina Schuster, Amel Chatti-kolsi, Mohamed Ali Triki, Mohieddine Ksantini, Andreas Leclerque

**Affiliations:** 1 Laboratory of Improvement and Protection of Olive Tree Genetic Resources, Olive Tree Institute, University of Sfax, Sfax 3038, Tunisia; 2 Laboratory of Biopesticides, Centre of Biotechnology of Sfax, University of Sfax, Sfax 3038, Tunisia; 3 Department of Biology, Technische Universität Darmstadt, 64289 Darmstadt, Germany

**Keywords:** bacteria, biological control, fungi, microbial diversity, *Olea europaea*

## Abstract

A wide array of bacteria and fungi are known for their association with pests that impact the health of the olive tree. The latter presents the most economically important cultivation in Tunisia. The microbial diversity associated with olive orchards in Tunisia remains unknown and undetermined. This study investigated microbial diversity to elucidate the microbial interactions that lead to olive disease, and the bio-prospects for potential microbial biocontrol agents associated with insect pests of economic relevance for olive cultivation in the Mediterranean area. Bacterial and fungal isolation was made from soil and olive tree pests. A total of 215 bacterial and fungal strains were randomly isolated from eight different biotopes situated in Sfax (Tunisia), with different management practices. 16S rRNA and ITS gene sequencing were used to identify the microbial community. The majority of the isolated bacteria, *Staphylococcus*, *Bacillus*, *Alcaligenes*, and *Providencia*, are typical of the olive ecosystem and the most common fungi are *Penicillium*, *Aspergillus*, and *Cladosporium*. The different olive orchards depicted distinct communities, and exhibited dissimilar amounts of bacteria and fungi with distinct ecological functions that could be considered as promising resources in biological control.

## 1. Introduction

The olive tree, *Olea europaea* L., belongs to the genus *Olea* within the Oleaceae family and Lamiales order. *Olea europaea* L., is one of the most important crops in the world. Olive production occupies an area of 11 million hectares worldwide, and the Mediterranean region is the most productive area, where 800 million olive trees are cultivated [1]. In Tunisia, the sixth largest producer of virgin olive oil [1,2], 1.7 million hectares is the surface dedicated to olive cultivation [3]. This culture is well adapted to the Mediterranean climate and is the most widely grown tree-crop species reported [4]. Olive cultivation plays a key role in Tunisians’ social and economic life.

It is well known that the olive groves are a relatively stable agroecosystem, but some insect pests, fungi, bacteria, and weeds, potentially harmful to the olive tree, cause crop yield losses and are responsible for economically significant levels of damage as well as significant losses in the olive yields reaching 100% in some cases [5,6]. In the Mediterranean basin, olive orchards are continuously damaged by major olive insect pests, such as the olive fly *Bactrocera oleae* (Diptera, Tephritidae), the olive moth *Prays oleae* (Lepidoptera, Hyponomeutidae), and the olive black scale *Saissetia oleae* (Homoptera, Coccidae), which may cause high economic losses [5]. The most prominent bacterial diseases are caused by *Pseudomonas savastanoi* (olive knot) and *Xylella fastidiosa* (olive quick decline syndrome). The latter is documented as being transmitted by xylem-fluid feeding insects, causing leaf scorching and dieback over an area of 8000 ha in Italy [7,8]. The most important fungal phytopathogens relevant for olive cultivation are *Spilocaea oleagina* (olive leaf spot); *Pseudocercospora cladosporioides Verticillium dahliae* (verticillium wilt); *Colletotrichum gloeosporioides* (anthracnose); *Fusicladium oleaginum* and *Mycocentrospora cladosporioides* (cercosporiosis) [9,10].

Nowadays, there is high agricultural productivity. The reduction in the negative impacts of agriculture and its activities, including pest management, is required. These impacts include nutrient pesticide contamination characterized by variable effectiveness and damaging effects on the ecosystems [11] and human health [12], as well as increased resistance to insects and diseases. The management of the olive pest and diseases are based on appropriate agronomical practices, such as the use of certified healthy material, solarization, balanced pruning, improvement of soil management, irrigation, and fertilization control [13]; and preventive chemicals, such as the use of cupric salts [14], dimethoate [15], thiophanate methyl [16], fosetyl-aluminum and benomyl [17]. Other control approaches include biological control, trapping methods, the release of sterile insects, and integrated control. 

Microbiota associated with the olive tree were suggested as an environmental alternative for controlling pathogens, and might be used as biological control agents (BCA). These microbial communities naturally associated with olive trees have been recognized for a long time as a source of promising biocontrol candidates that could be used for improving plant health [18,19,20,21,22].

In the present work, we emphasized the importance of the assessment of the bacterial and fungal communities associated with olive trees from different orchards in Sfax governorate (Tunisia). The microbial communities were identified and examined in relation to the olive orchards’ location and the period of isolation, with an emphasis on soil and insects. The study of genetic microbial diversity was investigated using molecular approaches.

## 2. Materials and Methods

### 2.1. Field Sites, Bacterial and Fungal Isolation

Sample collection was performed in 8 different olive groves located in distinct geographical zones in Sfax governorate (central-east Tunisia). The coordinates of the sample locations are presented in Table 1. Samples were randomly collected in the period ranging from December 2016 to December 2019. At each site, olive organs (i.e., flowers, mature fruit and leaves) infested by the olive moth larvae as well as 100 g of soil (from 10 cm of the upper soil layer) were sampled. Each sample was packaged in a sterile plastic bag (31 cm × 20 cm), transported to the laboratory under aseptic conditions and stored at 4 °C until bacteria and fungi were isolated.

Sampled insect cadavers were surface sterilized in a 70% ethanol solution for 1 min in a laminar chamber. The surface-sterilized insects were rinsed 3 times with sterile distilled water for 1 min. Each sample was ground in sterilized distilled water using a sterile micro-pestle. The soil samples were treated by dissolving 10 g in 90 mL of sterile distilled water and were mixed with a stomacher. Microorganisms were isolated by the plate dilution method [23,24]. Briefly, sample supernatants were serially diluted 10-fold and 100 µL of each dilution was spread onto Luria_Bertani agar media (LB: 10 g L^−1^ tryptone, 5 g L^−1^ yeast extract, 10 g L^−1^ NaCl, 15 g L^−1^ agar, pH = 7.0) and incubated at 32 °C for 24 h to 48 h for bacterial isolation, and potato dextrose agar media (PDA: 4 g L^−1^ Potato extract, 20 g L^−1^ Dextrose, 16 g L^−1^ Agar) was used for fungal isolation. PDA plates were incubated at 25 °C for 3 to 5 days. Bacterial and fungal colonies were purified by serial subcultivation on a fresh medium until pure cultures were obtained [23,24].

A total of 215 pure bacterial (96) and fungal (119) isolates were isolated after continuous sub-cultivation and were maintained on Luria_Bertani and potato_dextrose agar, respectively, for further investigation. For long-term storage, bacterial and fungal colonies were stored at −80 °C in glycerol (30%) and paraffin oil, respectively.

### 2.2. Molecular Identification of Isolates

Genomic DNA was extracted from the 215 microbial cultures using the DNeasy Mini kit (Qiagen^®^ GmbH, Hilden, Germany) for bacteria and the DNeasy Plant Mini kit (Qiagen^®^ GmbH, Hilden, Germany) for fungi, following the manufacturer’s instructions. Primers fD1 (5′-AGAGTTTGATCCTGGCTCAG) and rP2 (5′-ACGGACTTACCTTGTTACGACTT) for bacteria and ITS5 (5′-GGAAGTAAAAGTCGTAACAAGG-3′) and ITS4 (5′-TCCTCCGCTTATTGATATGC-3′) for fungi, and protocols used for PCR amplification of partial 16S rDNA gene and internal transcribed spacer ITS region were previously published [18,19]. PCR-amplified products were purified by QIAquick PCR Purification Kit (Qiagen^®^ GmbH, Hilden, Germany) applying the standard protocol provided by the manufacturer. Purified PCR products were Sanger sequenced on both DNA strands by a commercial sequencing facility using PCR primers (StarSEQ^®^ GmbH, Mainz, Germany). 

The obtained raw DNA sequence data were analyzed using the MEGA 6 program (http://www.megasoftware.net, accessed on 16 February 2017). 16S rDNA and ITS gene consensus sequences were generated for each bacterial and fungal isolate, respectively, and identified using the NCBI Genbank database (http://www.ncbi.nlm.nih.gov, accessed on 6 June 2018). The sequence information was used to match the most closely related taxonomically classified database entry by means of the BlastN algorithm available on the Genbank platform. The consensus sequence for each marker (ITS, 16S rDNA) was used as a query in BlastN searches for the most similar GenBank database entries. Results of at least 90% sequence coverage were sorted by decreasing sequence similarity percentage, and the following marker-specific cut-off values were applied: ITS 98% and 16S rDNA 97%. The exploration of microbial biodiversity was conducted at the genus level.

### 2.3. Statistical Analysis

The R-Studio program was used for all statistical analyses performed, including the Venn diagrams and heat maps [25].

## 3. Results

### 3.1. Microbial Community Composition

**Figure 1:**
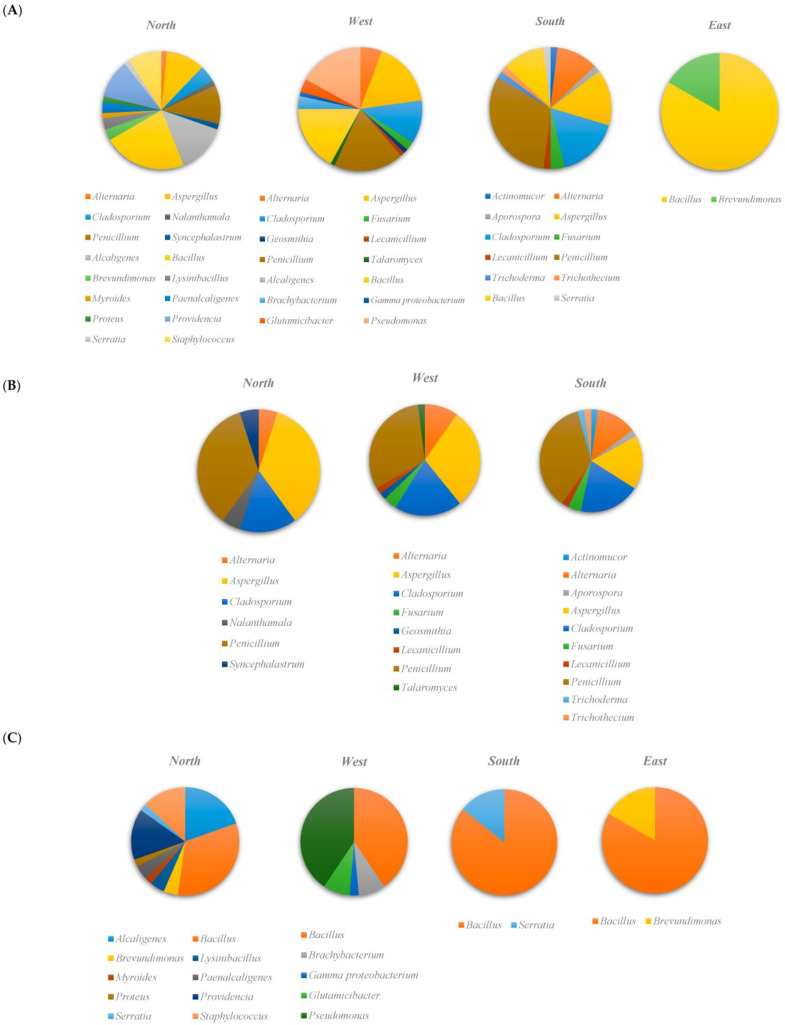
Microbial (**A**), fungal (**B**), and bacterial (**C**) community composition isolated from soil and insect (*Prays oleae*) at the genus level.

A total of 250 isolates were examined based on the 16S rDNA and ITS region sequences. In total, 86% (215/250) of the sequences in a BlastN search throughout the Genbank nucleotide database provided definitive assignment. The average query coverage and pair-wise sequence similarity scores for the bacterial and fungal sequences under analysis were 95%, 99%, and 93%, 98%, respectively. Purified bacterial and fungal strains included 96 bacteria originating from the insect (olive moth), and 119 fungal strains from soil and insect samples. The number of isolated fungi varied among the isolation matrices. Among them, 79 strains were from the insect species, while 40 strains were from the soil. A higher number of 16 bacterial and fungal genera was detected in Sfax North compared with Sfax West (14 genera), Sfax South (12 genera), and Sfax East (2 genera) (Figure 1A). Furthermore, Sfax North was characterized by the highest number of unique genera reaching 56.2% (9/16), which was not recovered from the other Sfax South, East, and West samples. In Sfax West, there were 46.1% (6/13) distinct genera, whereas in Sfax South, there were 38.5% (5/13).

Studying fungal isolates from insect and soil samples allowed for the identification of 14 fungal genera, 2 phyla (Ascomycota, Zygomycota), and 10 families (Cladosporiaceae, Cordycipitaceae, Didymosphaeriaceae, Hypocreaceae, Mucoraceae, Nectriaceae, Pleosporaceae, Syncephalastraceae, Trichocomaceae, *Trichothecium*) (Figure 1B). Among them, the genus *Penicillium* was the most strongly prevalent in three locations 33.6%, (40/119), followed by *Aspergillus* 25.2% (30/119) and *Cladosporium* 18.5% (22/119). Of the identified fungal strains present in the three locations (Sfax South, North and West), the genus *Cladosporium* was responsible for the most abundant species of *Cladosporium cladosporoides*, accounting for 7.6% (9/119). This latter was followed by *Aspergillus versicolor* 5.9% (7/119), *Alternaria alternata*, *Cladosporium ramotenellum*, *Penicillium copticola*, which had the same percentage of 5.0% (6/119), *Penicillium crustosum* 4.2% (5/119), and *Aspergillus ochraceus*, *Aspergillus tamarii,* and *Penicillium citrinum* with the same percentage of 3.4% (4/119).

Eight genera were found in only one location: *Talaromyces*, *Geosmithia*, in Sfax West, *Nalanthamala*, *Syncephalastrum* in Sfax North, *Actinomucor*, *Aporospora*, *Trichoderma*, and *Trichothecium* in Sfax South (Figure 1B). No fungal isolate was obtained from Sfax East.

Among the isolated bacteria, the *Bacillus* genus was the most frequently detected accounting for 85.7% (6/7) in Sfax South, 83.3% (5/6) in Sfax East, 40.5% (15/37) in Sfax West, and 32.6% (15/46) in Sfax North (Figure 1C). The *Bacillus* genus presented the highest frequencies of strain species (9), with *Bacillus subtilis* 24.4%, (10/41), *Bacillus cereus* 14.6% (6/41), *Bacillus licheniformis* 14.6% (6/41), *Bacillus thuringiensis* 12.2%, (5/41), and *Bacillus atrophaeus* 12.2%, (5/41) being the most detected. The insect colonization by *Alcaligenes*, *Providencia*, and *Staphylococcus* was detected only in the fields of Sfax North, and *Pseudomonas* in Sfax West.

### 3.2. Taxonomic Diversity According to the Isolation Location

**Figure 2:**
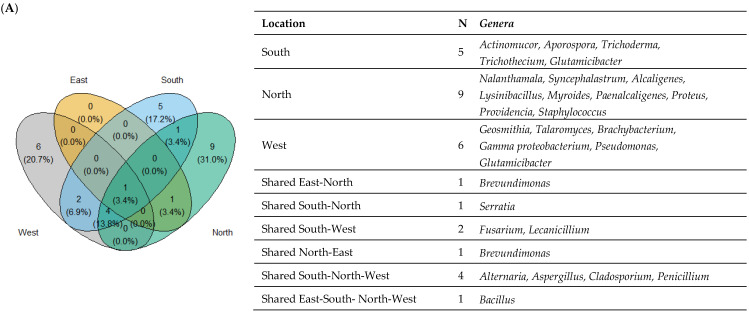

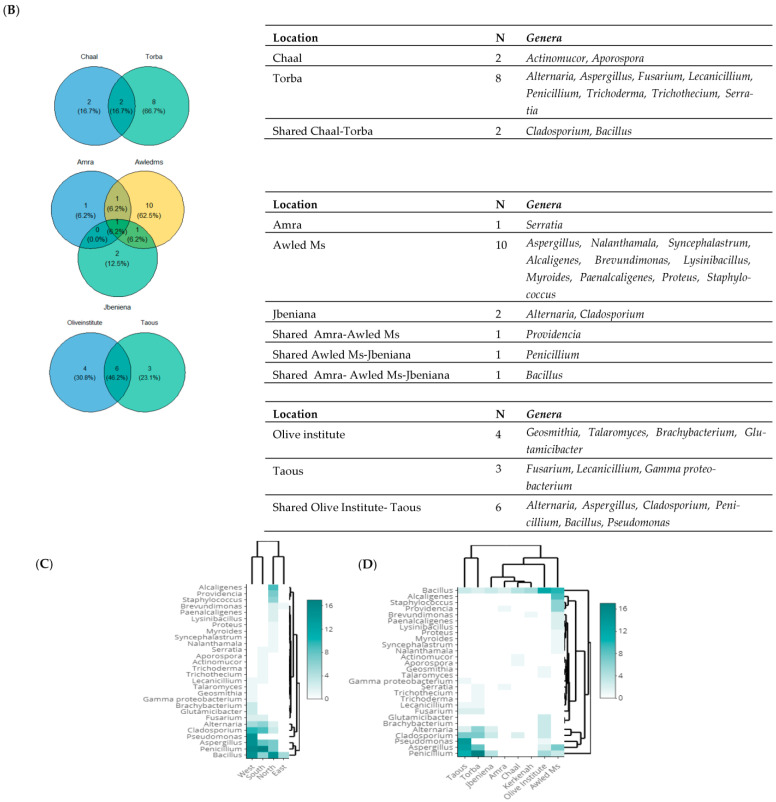
Taxonomic diversity according to the location of isolation from soil and insect (*Prays oleae*). (**A**) Venn diagram comparing the genera isolated from the east, south, north, and west. (**B**) Venn diagram comparing the genera isolated from the south, the north, and the west regions. The list of genera is presented at the right of each diagram. (**C**) Heat map of isolated genera from the east, south, north, and west. The row represents the tested isolate, and the column represents the region. The green color corresponds to the highest values, while the white color corresponds to the lowest one. (**D**) Heat map of isolated genera from the regions. N: Number of genera detected. The row represents the tested isolate, and the column represents the location. The green color corresponds to the highest values, while the white color corresponds to the lowest ones.

The shared and unique bacterial and fungal genera in different insects and soil samples from the various locations are shown in Figure 2A. *Bacillus* was the only genus present in the four studied locations. Additionally, *Brevundimonas* and *Serratia*, two distinct genera of bacteria, were also found in the overlapping east-north and south-north zones, respectively (Figure 2A). Moreover, the most abundant fungal genera isolates were *Alternaria*, *Aspergillus*, *Cladosporium*, and *Penicillium* which were shared in the south, north, and west of Sfax, representing 48.8% (105/215) of all isolates and 66.9% (105/157) of shared isolates. The isolates of *Fusarium* and *Lecanicillium* fungal genera were retrieved in the shared zone of east-west with a percentage of 3.8% (6/157).

Studying the eight fields, Ouled Msallem (Awled ms) displayed the highest detected genera being ten. The highest number of shared genera was observed between Olive Institute and Taous (six bacterial and fungal genera) including isolates of *Alternaria*, *Aspergillus*, *Cladosporium*, *Penicillium*, *Bacillus*, and *Pseudomonas*. The distinct *Bacillus* isolates were found in Awledms, Amra, and Jbeniana. Only Amra and Awled ms fields revealed the presence of *Providencia vermicola* species at a frequency of 85.7% (6/7) (Figure 2B).

Figure 2C depicts the two-dimensional hierarchical clustering of bacterial and fungal genera and the areas of their isolation. The color degradation revealed the number of isolates that were present at each specified position. This presentation made it possible to categorize strains according to where they were isolated. There were two recognized clusters. The first one presents the west and south isolates characterized by the dominance of fungal isolates, especially *Fusarium*, *Alternaria*, *Cladosporium*, *Aspergillus*, and *Penicillium* genera. However, bacterial strains predominated in the second cluster (Figure 2C). These findings are consistent with those obtained by clustering the isolation fields. In fact, at the genus level, *Bacillus* was present in all locations with a percentage of 29.3%, (12/41) in the Olive Institute (Figure 2D).

### 3.3. Taxonomic Diversity According to the Origin of Isolation

**Figure 3:**
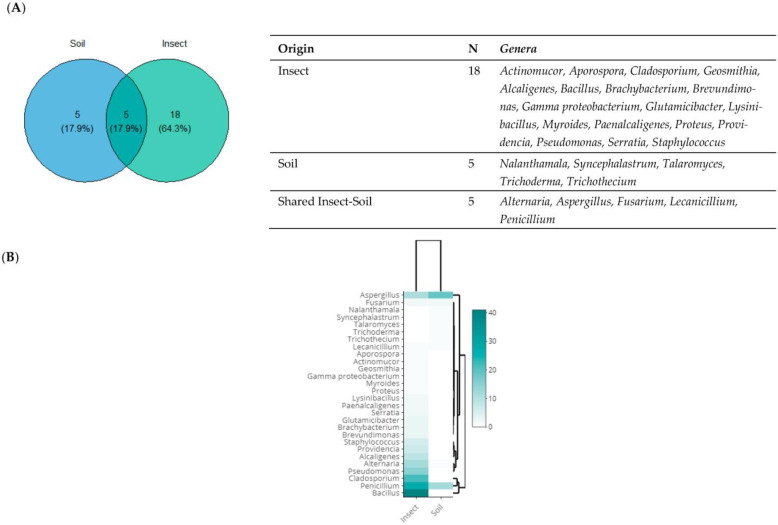
Taxonomic diversity according to the origin of isolation. (**A**) Venn diagram comparing the genera isolated from soil and insect (olive moth). The list of genera is presented at the right of the diagram. (**B**) Heat map of isolated genera from soil and insect. The row represents the tested isolate, and the column represents the origin of isolation. The green color corresponds to the highest values, while the white color corresponds to the lowest ones.

The Venn diagram displays the bacterial and fungal genera found in soil and insect samples (Figure 3A). In the intersection of the two areas under investigation, only fungal strains were found with a percentage of 17.9%, representing the *Alternaria*, *Aspergillus*, *Fusarium*, *Lecanicillium*, and *Penicillium* genera. At the species level, the shared zone was characterized by *Aspergillus ochraceus*, *Aspergillus tamarii*, *Aspergillus terreus*, *Aspergillus versicolor*, *Lecanicillium aphanocladii*, *Penicillium chrysogenum*, *Penicillium crustosum*, *Penicillium glabrum*, *and Penicillium pinophilum* with a rate of 25%, (9/36) of all the shared species. Both *Aspergillus* and *Penicillium* were isolated from soil and insect at relatively comparable levels (Figure 3B).

### 3.4. Taxonomic Diversity According to the Year of Isolation

**Figure 4:**
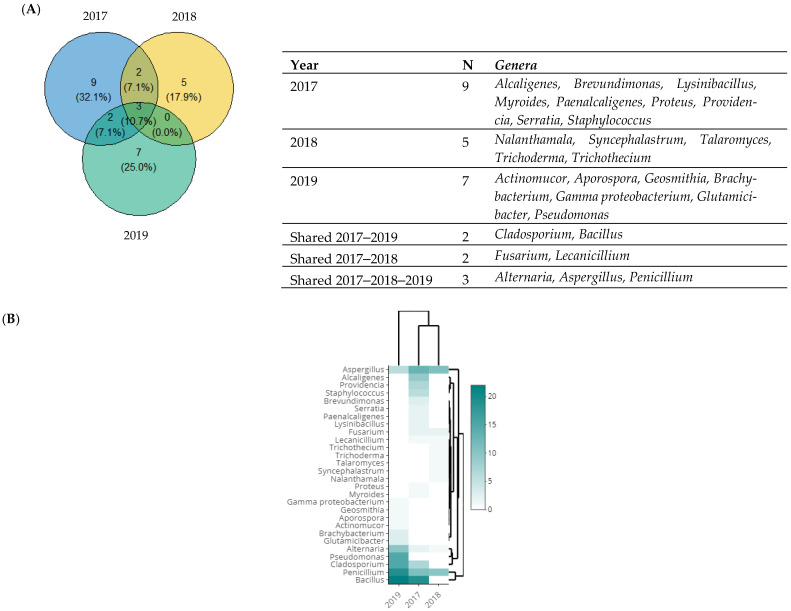
Taxonomic diversity according to the year of isolation. (**A**) Venn diagram comparing the genera isolated in 2017, 2018, and 2019. The list of genera is presented at the right of each diagram. (**B**) Heat map of isolated genera in 2017, 2018, and 2019. The row represents the tested isolate, and the column represents the year of isolation. The green color corresponds to the highest values, while the white color corresponds to the lowest ones.

A Venn diagram of fungal and bacterial genera, classified according to the year of their isolation is shown in Figure 4A. The *Bacillus* genus was the most prevalent in both 2019 and 2017, representing 50.0% (22/44) and 36.5% (19/52) (Figure 4B). This genus was followed by *Penicillium* detected in 35.8% (19/53) of the fungal samples, and *Pseudomonas* detected at a rate of 34.1% (15/44) in 2019. In addition, after the *Bacillus* genus, *Aspergillus* was the next most prevalent in 2017 at 36.1% (13/36), followed by *Alcaligenes* at 19.3% (9/52). Only three genera of fungi were identified in all three years, and these were *Alternaria*, *Aspergillus*, and *Penicillium*. With a rate of 18.6% (40/215), *Penicillium* was the most prevalent genus, followed by *Aspergillus* with a rate of 13.9% (30/215), and *Alternaria* was the third largest genus with a rate of 6.0% (13/215) of all isolated genera (Figure 4B).

## 4. Discussion

This study’s objective has been to understand the microbial communities in various olive orchards in the Sfax region of Tunisia, focusing on soil and insect pests. The location of the olive orchards and the period of isolation were also studied in relation to the microbial communities identified. The microorganisms examined in the olive groves in this study utilizing 16S rDNA and ITS gene analysis were bacteria and fungi, respectively. In line with earlier studies [26,27], 16S rRNA gene sequencing methods indicated Firmicutes, Proteobacteria, Actinobacteria, and Bacteroidetes as the most dominant phyla. These outcomes were in line with data from microbial communities linked to the root system of wild olives [28]. The endophytic microbiomes of healthy olive trees [29], microbiomes recovered from *Xylella*-infected plants [8,30], and bacteria isolated from dead insects found in olive tree orchards [23] all showed these four dominating phyla to be the most common. The variability in strain collection was also noted in a recent study that looked at the 16S rDNA sequencing of strains isolated from olives, olive pastes, and olive oils [31]. Actinobacteria and Firmicutes were found to have the highest percentage of isolates, followed by proteobacterial phyla [31]. Due to their resistance to UV radiation and desiccation, members of the identified phyla, particularly those belonging to the Actinobacteria and Firmicutes, were frequently detected in arid settings [20,32,33,34]. Additionally, they can survive in extreme environmental circumstances thanks to their capacity to produce spores [35]. These bacteria living in the olive tree are believed to strengthen their resilience and aid the host plant in surviving the abiotic challenges in the Mediterranean climate [20]. In our study, Firmicutes, which includes well-known antagonists and biocontrol agents, was the dominant phylum. According to Rache et al. a,b [36,37], Proteobacteria and Firmicutes represent a significant portion of the bacterial community. Proteobacteria typically live in nutrient-rich soils; hence, their presence in the soil is a sign of high soil nutrient content [38,39]. As for Actinobacteria, the Proteobacteria phylum is known as the most prevalent bacteria in the soil. It exhibits a wide range of physiological and metabolic characteristics, including the ability to produce extracellular enzymes and a wide range of secondary metabolites such as antimicrobial compounds [40,41]. 

Previous ITS microbiome studies indicated that Ascomycota and Basidiomycota were the most prevalent fungi in olive orchards [42]. These two fungal phyla belonged to the endophyte communities of above- and under-ground olive tree organs, as well as to the endophytic and epiphytic communities from the phyllosphere of the olive trees [18,43]. When investigating the fungi associated with the leaves, flowers, and fruits of olive trees at various phenological stages, Ascomycota and Basidiomycota were found in the phyllosphere and carposphere [44]. This variety of fungi was similarly isolated from dead insects that were gathered from Tunisian olive groves [24]. Vergine et al. reported the same conclusion for microbiota associated with the *X. fastidiosa*-resistant olive cultivar [30].

According to fungal genus variability, *Penicillium* and *Aspergillus* genera were the most collected samples, accounting for 33.6% (40/119) and 25.2% (30/119) of the strains, respectively. These results are in line with studies that emphasize *Penicillium* and *Aspergillus* as the most prevalent fungal species in nature (soils, plants, and agricultural communities), as well as the dominant genera in most ecosystems [5,45,46,47]. The varied agro-systems may have an effect on the diversity of microbes, particularly fungi. A conventional orchard would have less fungal variety than an organic or an integrated managed orchard, which creates a more biologically diverse and healthier environment [5]. With 36 different fungal species compared to 23 species in soil samples, insect samples had a higher species diversity.

The molecular identification of *Bacillus* based on bacterial genus was remarkable. *Bacillus* sp. was previously found in the endophytic communities of olive trees [26,28]. Our findings concur with those of Müller et al., who identified *Bacillus* as a fundamental member of the microbiota of the olive tree endosphere [26]. Moreover, *Bacillus* sp. was recovered from soil samples collected from olive groves [48]. The widespread occurrence of the genus *Bacillus* may be explained by its well-known capacity to produce a variety of antibacterial compounds. The synthesis of antimicrobial lipopeptide biosurfactants has already allowed the latter to be tested and authorized as biocontrol agents against various plant diseases [49,50,51,52,53].

In this study, we investigated the microbial communities regarding the type of soil/insect, year, or grove location, showing some specificity in some cases. In previous studies, the season was the main factor influencing the bacterial and fungal communities. In fact, bacterial and fungal endophytic populations of olive trees were also discovered to be impacted by seasonal fluctuations [8,19]. Gomes et al. reported that the highest quantity and diversity of bacteria and fungi were found in the spring, which may be the result of climate factors that encourage microbial growth or dispersal [18]. Additionally, the microbiome of an olive orchard was defined in soil-plant compartments, which compared bacterial communities from organic farming and conventional soil samples [27]. This later study revealed no discernible difference in soil bacterial diversity between the two systems, as previously reported by [54]. The bacterial populations in the phyllosphere of olive trees were discovered to be significantly influenced by the host cultivar, and, to a lesser extent, by plant organs [20].

The olive-associated microbiome has been described as a valuable source of microorganisms with potential as biocontrol and plant growth-promoting agents. In fact, members of the olive microbiome could represent new ecological solutions to be explored for diseases and plant management [55]. According to the literature, a number of biocontrol agents against diverse bacterial and fungal phytopathogens, as well as plant growth promoting agents from the olive tree-associated microbiome have been isolated and characterized. These microorganisms are well adapted to the olive environment and would perform better than those isolated from other sources. In our study, *Bacillus* and *Pseudomonas* species were the most detected bacteria. These species have been shown to greatly reduce *Pseudomonas savastanoi* pv. *savastanoi* causing olive knot disease [20,56,57]. In addition, Cheffi et al. [52] reported the isolation, characterization and screening of the root microbiome of olive trees against numerous fungal pathogens that have led to the identification of the *Bacillus velezensis* OEE1, with plant growth promotion abilities and strong activity against major oomycete and fungal pathogens including *Fusarium solani*. The authors suggest that this strategy could provide a direct approach to enrich the biocontrol toolbox against recalcitrant *Phytophthora* and *Fusarium* species. In our previous study [19], we selected strains of *Aspergillus pseudodeflectus* and *Lecanicillium aphanocladii*, isolated from dead insect pests collected from Tunisian olive groves, and showed their ability to protect olive orchards due to their insecticidal activity against the lepidopteran pests. Ben Amira et al. [58] reported the use of *Trichoderma harzianum* (*Ths97*) on olive trees against *Fusarium* root rot disease caused by *Fusarium solani*. On olive trees, this fungus developed a strong protective role against root infestation by the phytopathogenic strain, under both curative and preventive treatments. The use of endophytic microorganisms and an endophytic microbial consortium of different bacterial and/or fungal species originating from the olive tree microbiome were also suggested to be useful strategies for protecting the olive tree against diseases [59,60,61].

## 5. Conclusions

This work highlights the high microbial biodiversity that has been observed in olive orchards in Sfax, Tunisia, and it also confirms that some bacterial and fungal species could be generally considered distinctive of the strain’s origin, isolation location, and year of isolation. *Penicillium*, *Aspergillus*, and *Cladosporium* were among the fungal taxa most frequently associated with the ecosystems of olive trees, while *Actinomucor*, *Aporospora*, *Geosmithia*, *Nalanthamala*, and *Syncephalastrum* were less frequently encountered. We discovered that most of the isolated bacteria, including *Bacillus*, *Alcaligenes*, *Providencia*, and *Staphylococcus*, are typical of the olive ecosystem. Future research on the biotechnological and biocontrol capabilities of these bacteria and fungi may be pursued, considering the possibility that they are crucial to the survival of olive trees in Mediterranean climates.

## Figures and Tables

**Table 1:** Sampling sites and their coordinates.

Sampling Sites	Coordinates		Location
	Longitude	Latitude	
Amra	10°53′09″ E	34°59′07″ N	Sfax North
Ouled Msallem	10°49′19″ E	34°59′31″ N	Sfax North
Jbeniena	10°54′16″ E	35°03′10″ N	Sfax North
Torba	10°36′47″ E	34°55′17″ N	Sfax South
Olive Institute	10°43′59″ E	34°44′04″ N	Sfax West
Taous	10°27′33.6″ E	34°50′56.9″ N	Sfax West
Chaal	10°19′19″ E	34°27′27″ N	Sfax South
Kerkennah	11°00′16″ E	34°39′09″ N	Sfax East

## Data Availability

Raw data that support the findings of this study are available from the corresponding authors, up on reasonable request.
